# Study on the Effect of the Plunging Depth of Stirring Pin on the Performance of 6061-T6 Aluminum Alloy Refill Friction Stir Spot Welded Zone

**DOI:** 10.3390/ma18163921

**Published:** 2025-08-21

**Authors:** Di Jiang, Igor Kolupaev, Hongfeng Wang, Xiaole Ge

**Affiliations:** 1 College of Mechanical and Electrical Engineering, Huangshan University, Huangshan 245041, China; wanghnfeng@163.com (H.W.); gxl_ntu_khpi@163.com (X.G.); 2 Department of Materials Science, Kharkiv Polytechnic Institute, National Technical University, 61002 Kharkiv, Ukraine; igor.kolupaev@gmail.com

**Keywords:** refill friction stir spot welding, plunging depths of stirring pin, ultimate lap shear force, aluminum alloy

## Abstract

In this study, under varying PDSP (plunging depths of stirring pin) and process parameters, refill friction stir spot welding tests were performed on 6061-T6 aluminum alloy, relying on a stirring tool with a 12 mm sleeve diameter and an 8 mm stirring pin diameter. The results manifested the internal defects in the weld zone when PDSP was 0, notwithstanding the alterations in process parameters. However, these flaws disappeared when PDSP was 0.5 mm and 1 mm. In the weld zone, PDSP exerted a dramatic effect on the internal metal flow state, particularly the curvature of the “Hook” shape and the width of the bonding ligament. It changed the downward bending of the ‘Hook’ into an upward one, influencing the fracture behavior of the weld zone and elevating the ULSF (ultimate lap shear force) by up to 20% (PDSP = 0.5 mm, welding speed = 30 mm/min, rotation speed is 1200 rpm). Besides, the PDSP intensified the PAZ (pin affected zone) pressure, induced more metal flowing into the SAZ (sleeve affected zone), thus reinforced the SAZ-TMAZ(thermomechanically affected zone) bonding strength, and upgraded the region’s microhardness. In summary, the PDSP is commendable for bolstering the weld zone’s performance, but excessively large PDSP values incur drastic indentations in the PAZ, which diminish the ULSF.

## 1. Introduction

Refill friction stir spot welding (RFSSW) has been capturing immense attention from aerospace, automotive, lightweight manufacturing, and other sectors. It relies on the high-speed rotation friction of the coaxially fitted stirring pin and stirring sleeve to generate heat. During spot welding, the stirring sleeve with an internal cavity is typically inserted into the material, plasticizing it and allowing it to flow into the internal cavity. In the backfilling stage, the stirring pin gradually extrudes the plasticized metal inside the stirring sleeve to form a weld nugget, resulting in a smooth and flat spot weld. The entire spot-welding process does not involve melting the material or adding extra material, which provides significant technical advantages when welding temperature-sensitive lightweight alloys. With the rocketing application of lightweight high-strength materials (e.g., aluminum alloys) in structural connections, traditional fusion welding processes confront a myriad of challenges, as exemplified by unstable joint quality, property degradation in the heat-affected zone, and welding spatter, which hamper the compliance with the joint requirements on high-performance structural components. RFSSW, boasting its merits of not melting material during the joining process, low thermal input, dense joint microstructure, and robust joint strength, has been established as a pivotal technical alternative [1,2].

This process considerably appeals to researchers thanks to its distinct benefits. Current research chiefly focuses on the microstructural evolution in the weld zone, process parameters, and the structural design of the stirring tool. Silva et al. [3] delved into the effects of rotational speed (1500~3100 Rpm), welding speed (3.0~4.5 mm/s), and plunge depth (1.9~2.3 mm) of the shoulder on the tensile-shear strength of 6082-T6 aluminum alloy (2 mm), the best ULSF (8.5 ± 0.3 KN) was obtained under parameters of rotational speed at 2700 Rpm, penetration depth at 2.1 mm, and welding speed at 3.9 mm/s. As demonstrated by the results, the plunge depth imposed the most prominent influence on shear load. However, it also indicates that greater penetration depth does not necessarily result in higher strength, which is aligned with findings from other studies [4,5]. Jiang D et al. [6,7,8] examined the influence of tool diameter, the ratio between probe and shoulder, and the process parameters on weld zone performance. They concluded that, in the event of a 6 mm probe diameter, a higher rotational speed and lower welding speed contribute to bolstering tensile-shear strength. Prolonging the probe diameter to 8 mm further enhances the ULSF. When spot welding 6061-T6 aluminum alloy with a thickness of 3 mm, the ULSF of 14.7 kN can be achieved, which amplifies the sensitivity to process parameters. When adjusting the diameter ratio between pin and sleeve—for instance, involving a 13.5 mm sleeve diameter and a 10 mm pin—the performance surpasses that with a 9 mm pin, and turns less sensitive to process parameters.

In addition, some scholars surveyed how the welding time affects the tensile-shear strength of RFSSW joints, and its influence on the final mechanical performance was found relatively insignificant [9,10]. Shen Z et al. leaned on a grooved stirring tool to explore material flow, stir behavior, and liquation cracking during the RFSSW process of both similar and dissimilar aluminum alloys [11,12,13]. The results indicate that grooved stirring tools are more effective than threaded ones for spot welding dissimilar materials, although localized metal melting occurs. When spot welding 6022-T4 aluminum alloy and 7075-T6 aluminum alloy with a thickness of 2 mm, an optimal ULSF of 4.19 kN was achieved at a welding time of 4.5 s, further demonstrating the technological advantages of RFSSW. When using FSSW to weld 2 mm thick 2024 and 7075 aluminum alloys, an optimal ULSF of 4.06 kN was achieved when the welding time exceeded 45 s, with the welding time increased by tenfold [14]. Likewise, Ji S et al. [15] scrutinized the impact of the stir tool’s geometry and summarized that tools with grooves perform more favorably in boosting the overall performance of the weld zone. It also increases the diameter of the weld nugget, but it also clearly points out that this method of increasing the weld nugget diameter is significantly inferior to enlarging the diameter of the stirring tool.

Additionally, Gale D et al. [16] studied the performance comparison between RFSSW and resistance spot welding (RSW) for 6061-T4 aluminum alloy. The results showed that the average grain size within the spot weld zone was 2.58 μm for RFSSW and 64.83 μm for RSW. RFSSW weld nuggets were consistently 5–10 Vickers harder than RSW weld nuggets. RFSSW outperformed RSW in quasi-static tensile strength, with RFSSW joints being between 16 and 73% stronger than RSW joints, and 2600% more cycles before fracture. Zhao Y et al. [17] controlled the heat input to the spot-welding zone by adjusting the rotational speed of the stirring tool at different stages, successfully completing variable-speed refill friction stir spot welding (V-RFSSW) based on 7B04-T74 aluminum alloy. The results showed that under the same rotational speed conditions, the average hardness and tensile shear strength of V-RFSSW joints were higher than those of RFSSW joints.

The existing body of research has primarily focused on enhancing the tensile-shear performance of the spot-welded zone, which is directly related to the fact that this zone is mainly subjected to tensile-shear loads in practical applications. One common approach is to improve performance by modifying the diameter and geometry of the stirring tool and optimizing process parameters to obtain a more favorable processing window. While these methods are effective, they often encounter technical limitations that hinder further improvements in joint performance.

Notably, previous studies consistently point out that improving the metal flow behavior within the weld zone is beneficial. Building on this insight, the present study analyzes the process of RFSSW and introduces a simple yet effective method: driving the stirring pin downward into the upper sheet during welding. This is done without altering the tool diameter, geometry, or welding duration. The aim is to enhance the metal densification and forging pressure in the weld zone. Furthermore, this work investigates the influence of the pin plunge depth on the joint’s mechanical performance, offering a new approach for improving spot weld quality. This method also lays the groundwork for future research, where it could be combined with other strategies to further expand the development of RFSSW techniques.

## 2. Experimental Materials and Methods

**Figure 1:**
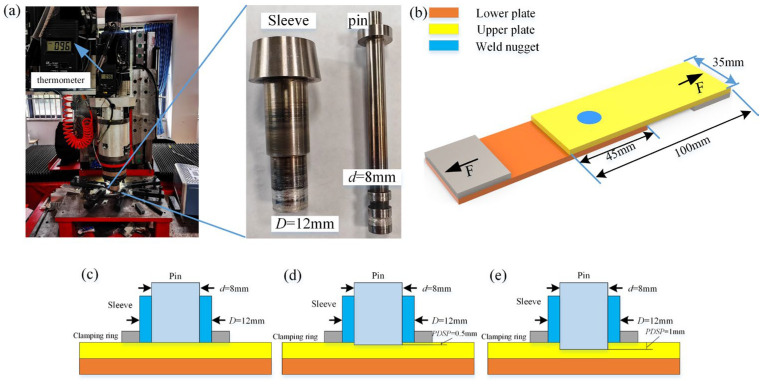
Equipment and process schematic diagrams (**a**) RFSSW equipment, (**b**) Diagram of tensile shear, (**c**) PDSP = 0, (**d**) PDSP = 0.5 mm, (**e**) PDSP = 1 mm.

This study hinges on 3 mm-thick 6061-T6 aluminum alloy as the test material, which is distinguished by superior plasticity and mechanical properties. Its specific performance parameters are encapsulated in Table 1 and Table 2. This data comes from China Guangdong Province, Guangzhou City Jinsheng Metal Co., Ltd., Guangzhou, China, which is a manufacturer of metal materials and has been verified by the author’s actual measurements; its measurement method is consistent with the performance testing method of the subsequent spot-welding zone. The RFSSW equipment produced by China Beijing Safostar Company, Beijing, China, was adopted for welding, with a stirring pin diameter of 8 mm and a stirring sleeve diameter of 12 mm, as illustrated in Figure 1a.

In practical applications, the surface of RFSSW generally needs to be relatively flat, which means that the PDSP cannot be too large, not exceeding 20% of the thickness of the welded completed plate. The main meaning of PDSP refers to the fact that during spot welding, the stirring pin protrudes from the stirring sleeve, whereas in the conventional RFSSW process, the stirring pin aligns with the stirring sleeve at the completion of spot welding. In this study, PDSP is achieved by altering the equipment control program. Therefore, the PDSP was set to 0 mm, 0.5 mm, and 1 mm, with their specific process forms depicted in Figure 1c–e, respectively. In pursuit of better testing on the ULSF of the spot weld zone, support plates were positioned on both sides of the sheet, as shown in Figure 1b. The tensile shear speed was kept constant at 1.5 mm/min and complied with ISO 18785-5:2018 [17], Friction stir spot welding—Aluminium, Part 5: Quality and inspection requirements, MOD standard. For each process parameter, the ULSF test was conducted five times, with the average value taken. To better study the effect of PDSP on the spot welding zone, the rotational speed was kept constant at 1200 rpm, with actual rotational speed fluctuations not exceeding 0.5% under servo motor control, radial oscillation of the stirring tool not exceeding 0.03 mm, and welding speed limited by equipment performance, set at 30 mm/min, 40 mm/min, and 50 mm/min. The welding speed refers to the axial movement speed of the stirring sleeve, which remains unchanged during the penetration stage and refilling stage. The dwell time was set to 2 s, the welding pressure was kept constant at 7 kN, the maximum torque of the rotating main shaft was 25 Nm, and the diameter of the clamping ring was 25 mm. To ensure relatively consistent initial conditions for each spot weld, a temperature sensor was placed 50 mm above the clamping ring to detect temperature changes on the outside of the entire stirring tool, and wind cooling was used to stabilize it at 100 ± 5 °C, as shown in Figure 1a.

## 3. Experimental Results

### Appearance of the Spot Welding Zone

**Figure 2:**
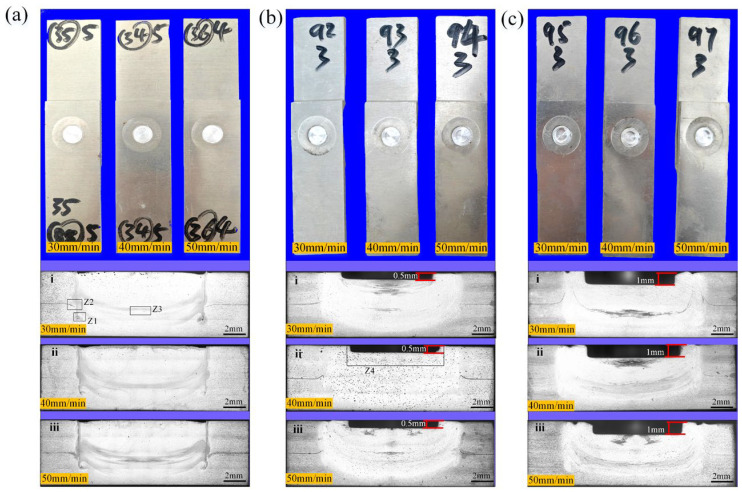
Spot weld zone and macroscopic cross-sectional morphology. under different processes (**a**) PDSP = 0 (**b**) PDSP = 0.5 mm (**c**) PDSP = 1 mm (welding speed: i = 30 mm/min, ii = 40 mm/min, iii = 50 mm/min).

Figure 2 reveals desirable forming in the spot-welding zone under varying insertion amounts. On the cross-section of the spot-welding zone, a groove can be observed, with a depth conforming to the insertion amount of the stirring pin, and its width is basically the same as the stirring pin’s diameter. This can be explained by the stirring pin being inserted below the spot-welding plane. From the changes in welding speed, it appears that there is no significant change in the macroscopic morphology of the spot weld zone.

**Figure 3:**
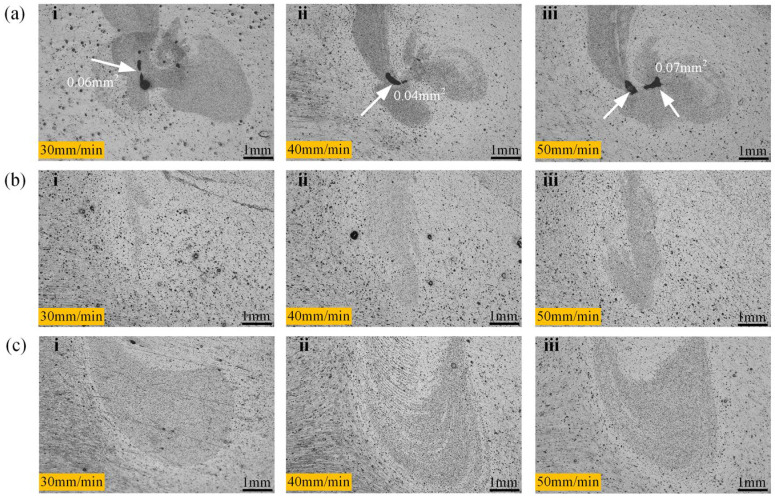
Microscopic morphology of internal defects in spot welding zone. (The sampling relative position as shown in Figure 2, Z1). (**a**) PDSP = 0 (**b**) PDSP = 0.5 mm (**c**) PDSP = 1 mm (welding speed: i = 30 mm/min, ii = 40 mm/min, iii = 50 mm/min).

When the PDSP = 0, evident void defects can be noticed in the “droplet zone,” as plotted in Figure 3a, which persist in spite of changes in the welding speed. Nevertheless, when PDSP ≠ 0, no such void defects are present, and the area of the droplet zone expands with the enlarging plunge depth, as illustrated in Figure 3b,c.

This suggests that the droplet zone is the most difficult zone for metal flow within the weld region. During the welding process, the volume displaced by the stir sleeve entering the material should, in theory, be fully compensated by the upward movement of the stir pin. However, practically, the shoulder inevitably displaces the surrounding metal outward as it plunges, causing some material to escape from the weld zone. Hence, the cavity originating between the pin and the sleeve may not be thoroughly filled and may instigate void formation during refilling.

In the case of PDSP ≠ 0, the stir pin pushes further into the weld zone, exerting extra pressure on the surrounding material. This notably increases the quantity of metal engaged in the refilling process, fostering the elimination of void defects. Moreover, a greater pin plunge depth arouses stronger compression, which in turn enlarges the area by forcing more material into the droplet zone. Therefore, the presence of PDSP is beneficial for a defect-free weld region.

**Figure 4:**
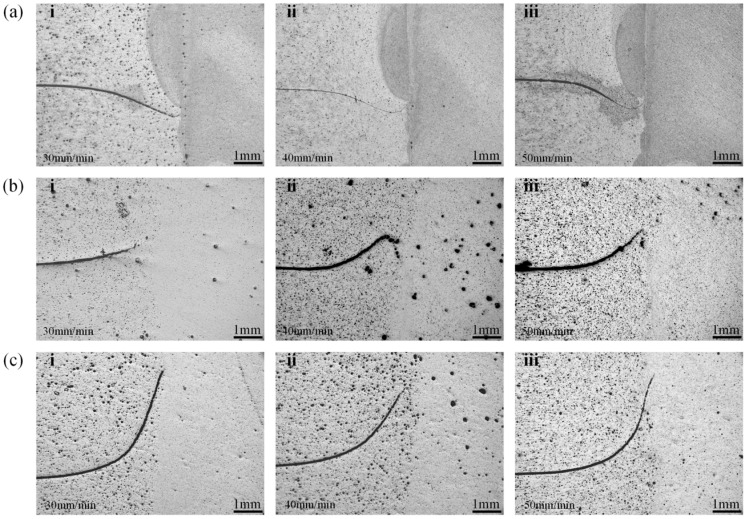
Morphology of the ‘Hook’ in spot welding zone. (The sampling relative position as shown in Figure 2, Z2). (**a**) PDSP = 0 (**b**) PDSP = 0.5 mm (**c**) PDSP = 1 mm (welding speed: i = 30 mm/min, ii = 40 mm/min, iii = 50 mm/min).

In RFSSW research, the morphology of the “Hook” feature has long been a focal point for studying the metal plastic flow and the bonding between upper and lower sheets [18,19,20]. When PDSP = 0, the “Hook” consistently bends downward across all welding speeds, as shown in Figure 4a, which contradicts many previous findings [3,18,19,20]. Such a discrepancy is believed to stem from the larger diameter of the stirring tool used in this study, which results in a thicker sleeve and a greater volume penetrating the material. In this case, the stirring sleeve will cause more metal to bend downward, which is very similar to the results of the author’s other studies [6,7,8]. Consequently, the stronger force exerted by the sleeve during plunging causes the “Hook” to bend downward.

In contrast, when PDSP ≠ 0, the “Hook” bends to the opposite direction, as visualized in Figure 4b, and Figure 4c unveils that the degree of upward bending ascends with the enlargement of PDSP. This behavior can be ascribed to the metal flow dynamics during the refilling stage. With pin plunge, metal within the weld zone is forced to flow outward. The “Hook” typically forms adjacent to the sleeve, and a fully inserted sleeve lies below the “Hook”. When the sleeve retracts upward during refilling, a temporary cavity is created beneath it, and the pin’s downward movement ensues to compress the metal to refill this cavity.

Because the PDSP causes the volume of the pin-pushed metal to exceed the volume of the cavity left by the retracting sleeve, excessive metal flows into the space beneath the sleeve. This provokes deformation of the surrounding metal and initiates the “Hook” morphology, shifting from downward to upward bending. A greater PDSP increases the quantity of displaced metal and intensifies the upward bending.

The study also observed that the “Hook” morphology appears to be independent of welding speed. This implies that the formation of the “Hook” is not influenced by the upward movement of the sleeve during the refilling stage. Otherwise, as welding speed accelerates and the sleeve retracts more swiftly, dramatic variations would be expected in the “Hook” morphology. The absence of such changes affirms the proposed explanation for the “Hook” formation, reinforcing its validity and consistency with the observations.

**Figure 5:**
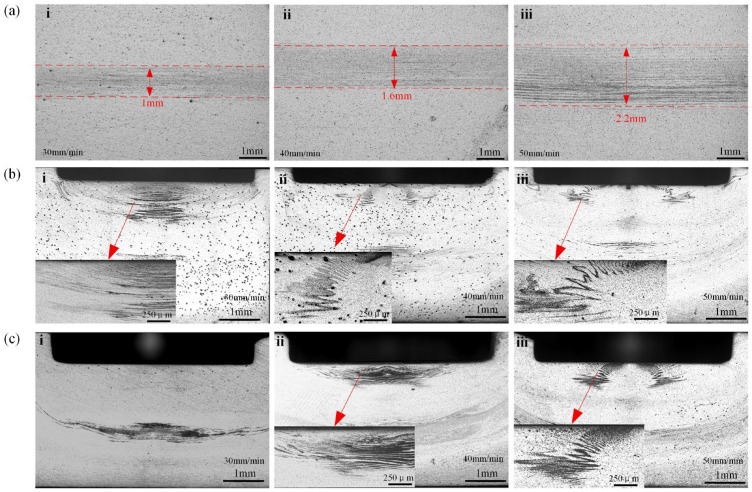
Morphology of the bonding ligament in spot weld zone (**a**) PDSP = 0 (Sampling relative position as shown in Figure 2, Z3) (**b**) PDSP = 0.5 mm (Sampling relative position as shown in Figure 2, Z4) (**c**) PDSP = 1 mm. (Sampling relative position as shown in Figure 2, Z4) (welding speed: i = 30 mm/min, ii = 40 mm/min, iii = 50 mm/min).

Bonding ligaments are frequently identified in the spot weld zone and are derived from the incomplete disruption of the interface between upper and lower sheets. They act as a clear degree indicator of metal fragmentation within the weld zone. In the event of PDSP = 0, the bonding ligament typically arises at the center of the PAZ and widens with the accelerating welding speed. The reason lies in the insufficient mechanical fragmentation caused by the central region that is directly contacted by the stirring tool. As the welding speed is expedited, the shortened stirring time engenders a wider bonding ligament, as illustrated in Figure 5a. When the PDSP ≠ 0, the same trend persists, but a distinctive serrated line appears at the upper boundary of the PAZ. This feature is supposed to derive from the same mechanism as the bonding ligament, yet in this case, its origin is the upper surface interface of the top sheet. The pin’s downward movement induces more intense material flow and causes the upper sheet interface to intrude into the weld region. With the acceleration of the welding speed, the depth of this intrusion tapers off, as longer stirring durations foster deeper penetration, as depicted in Figure 5c. When the PDSP further enlarges, the enhanced compressive fragmentation thoroughly disrupts these original upper-sheet interfaces, rendering the serrated lines unobservable. With a faster welding speed and shorter stirring time, the serrated features begin to reappear, which explains their invisibility at the lowest welding speed, according to Figure 5c. These findings demonstrate that probe plunge profoundly impacts the internal material flow behavior in the weld zone, especially within the PAZ.

**Figure 6:**
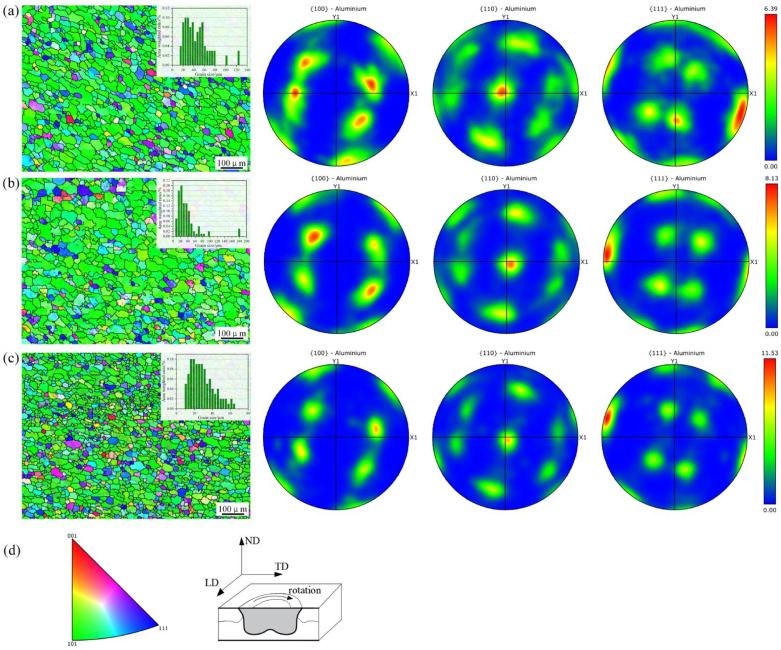
Grain distribution in the PAZ (**a**) PDSP = 0, (**b**) PDSP = 0.5 mm, (**c**) PDSP = 1 mm, (**d**) Testing orientation and corresponding direction.

At the same welding speed, the grain orientation within the PAZ remains consistent across different PDSP. However, the orientation intensity is augmented with PDSP, while the average grain size progressively shrinks—measured at 45.8 μm, 37.2 μm, and 29.5 μm, as plotted in Figure 6. The scanning resolution of these EBSD images is no less than 92%, with a scan zone of 800 μm in width and 600 μm in height.

This substantiates that, although not altering the overall flow pattern of the metal in the PAZ, changing the PDSP intensifies the compressive deformation impetus for grain refinement. As a result, despite the similar grain orientation across different PDSP, orientation intensity is boosted by the stronger compressive force at greater PDSP. Hence, the highest plunge depth yields the finest grains and the strongest orientation intensity.

**Figure 7:**
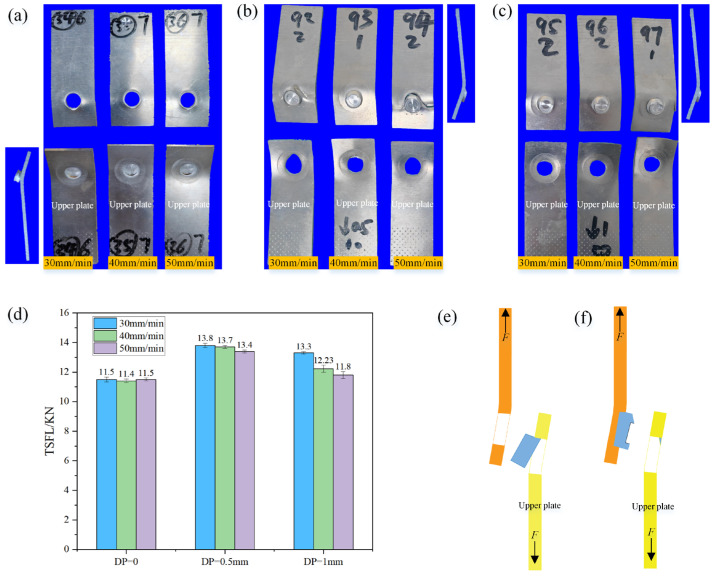
Fracture morphology and tensile-shear performance of weld zone under different process parameters (**a**) PDSP = 0, (**b**) PDSP = 0.5 mm, (**c**) PDSP = 1 mm. (**d**) ULSF under different PDSP values (**e**) Schematic of fracture mode at PDSP = 0 (**f**) Schematic of fracture mode at PDSP ≠ 0.

**Figure 8:**
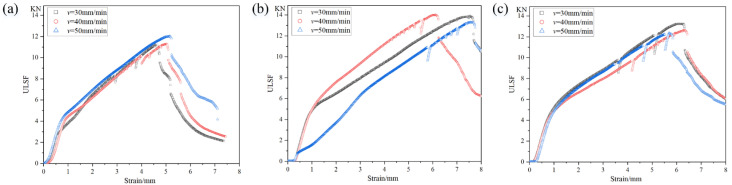
Stress-strain curve of weld zone under different process parameters (**a**) PDSP = 0, (**b**) PDSP = 0.5 mm, (**c**) PDSP = 1 mm.

When the PDSP ≠ 0, the effect of welding speed on ULSF increases with the increase in penetration depth. The fracture morphology of the spot weld zone is basically a partial protrusion fracture, with the weld nugget completely pulled out from the upper plate and partially cut off at the front end, as shown in Figure 7b,c, and its specific fracture diagram is shown in Figure 7f [21]. At PDSP of 0.5 mm, the highest ULSF is obtained. From the stress-strain curve shown in Figure 8, it can be seen that these fractures are mostly plastic fractures. During the plastic deformation stage, the study notices a sudden drop in tensile force followed by a continued increase, and this phenomenon becomes more frequent as PDSP increases. Observing the actual stretching process, this is due to the presence of pressure diffusion welding around the spot weld area between the upper and lower plates. A larger PDSP generates greater pressure, resulting in stronger pressure diffusion welding. During the stretching process, it suddenly breaks, causing a sudden drop in tensile force, but this does not affect the final ULSF.

When the PDSP = 0, it is a complete protrusion fracture, with the weld nugget completely pulled out from the lower plate, as shown in Figure 8a, but there is also severe tearing between the upper plate, and its specific fracture diagram is shown in Figure 7e. Analysis suggests that this is due to the existence of stirring pin penetration depth, which helps to increase the pressure between SAZ and TMAZ, reduces the risk of defects, and changes the shape of ‘Hook’, all of which directly affect the position and direction of initial cracks. This behavior leads to some defects in the SAZ of the lower plate when there is no PDSP, reducing its strength and making it easy for initial cracks to form here, and at this time, the ‘Hook’ also bends towards the lower plate, further guiding the initial crack to grow downward, ultimately causing the weld nugget to be completely pulled out from the lower plate. When there is a stirring pin penetration depth, there are no defects inside the spot weld zone, and the ‘Hook’ bends towards the upper plate, causing the crack to extend along the ‘Hook’ upward from the interface between the upper and lower plates, ultimately leading to the weld nugget being pulled out from the upper plate. Meanwhile, due to the presence of PDSP, PAZ has a groove, which makes the front end of the weld nugget easy to be cut off when it is about to be completely pulled out, resulting in the ULSF level at PDSP of 1 mm being lower than the stirring pin penetration depth of 0.5 mm. Overall, the ULSF of the spot weld zone is significantly higher when there is PDSP, with a maximum increase of 20%. Without stirring pin penetration depth, due to some internal defects, and the change range of ULSF under different welding speeds is very small, indicating that internal defects are the main factor affecting ULSF at this time, as shown in Figure 7d.

**Figure 9:**
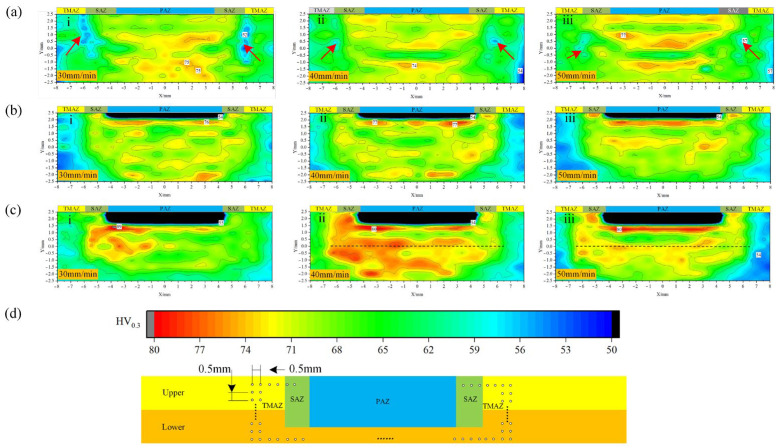
Microhardness distribution in weld zone under different process parameters (**a**) PDSP = 0, (**b**) PDSP = 0.5 mm, (**c**) PDSP = 1 mm. (**d**) Microhardness test locations and corresponding scale (welding speed: i = 30 mm/min, ii = 40 mm/min, iii = 50 mm/min).

According to the hardness measurement method specified by ASTM E384 standard, a Vickers hardness tester is used for hardness testing. The square-based pyramid indenter is pressed into the material surface under a load of 0.3 kg and held for 10 s. The hardness value is obtained by measuring the length of the diagonals of the indentation using an optical measuring instrument. Each sample is measured three times, and the average value is taken as the final result. The obtained hardness distribution is shown in Figure 9, where the measurement locations and intervals are illustrated in Figure 9d. In the absence of PDSP, a low-hardness region is spotted at the interface between the TMAZ and the SAZ, which designates the internal defects previously identified in the weld zone, as signified by the arrows in Figure 9a; the hardness of the base material is 90 HV_0.3_. In terms of average hardness, the values remain nearly constant across different welding speeds, i.e., 66.7 HV_0.3_, 67.0 HV_0.3_, and 66.7 HV_0.3_. Upon the PDSP application, this low-hardness region at the TMAZ-SAZ interface disappears, and a distinct high-hardness layer emerges on the upper side of the PAZ. The thickness of this hardened layer extends along with the acceleration of welding speed, as shown in Figure 9b,c. Such an effect is owing to the compressive deformation intensified by the pin’s downward movement, which enhances local strain hardening near the PAZ.

At the PDSP = 0.5 mm, the average hardness values slightly increase with welding speeds: 68.1 HV_0.3_, 68.3 HV_0.3_, and 68.6 HV_0.3_. However, when the PDSP = 1 mm, the average hardness dwindles as the welding speeds increase to 71.6 HV_0.3_, 69.2 HV_0.3_, and 66.7 HV_0.3_, respectively. This trend closely mirrors the variation in ULSF, indicating that, while enhancing local compression and hardening the upper PAZ layer, a faster welding speed shortens the overall welding time. As a result, the shorter stirring duration is to the detriment of the tool’s ability to fragment and mix the material, leading to a decline in average hardness with higher welding speeds.

## 4. Conclusions

This study focused on RFSSW of 3 mm-thick 6061-T6 aluminum alloy under varying PDSP, and its principal conclusions are elucidated as follows:

(1) By leveraging a stirring tool with a 12 mm stir sleeve diameter and an 8 mm stir pin diameter, welding was carried out at a rotational speed of 1200 rpm and welding speeds of 30 mm/min, 40 mm/min, and 50 mm/min, when PDSP = 0 mm PDSP = 0.5 mm, and PDSP = 1 mm, respectively. The results reveal that applying PDSP is capable of slashing internal defects within the weld zone.
(2) PDSP prominently influences the internal metal flow behavior of the weld zone, particularly the bending morphology of the “Hook” and the width of the bonding ligaments—these changes exert direct impact on the ULSF. With PDSP = 0.5 mm, welding speed of 30 mm/min, the ULSF rises by up to 20%, yet the ULSF at PDSP = 1 mm lower than that of PDSP = 0.5 mm. Therefore, according to the results of this study, PDSP should not exceed 10% of the spot weld thickness. In both cases, ULSF tapers off with the accelerating welding speed.
(3) The presence of PDSP strengthens the compressive force in the PAZ, which induces more metal to flow into the SAZ. This intensifies the interfacial pressure between the SAZ and the TMAZ, thereby reinforcing their bonding strength. This is further corroborated by the higher microhardness observed in these zones. Consequently, PDSP is conducive to bolstering the weld zone’s overall performance. However, excessive PDSP gives rise to deep grooves in the PAZ, which may abate the load-bearing capacity of the joint. At the same time, when PDSP = 1 mm, greater axial pressure can result in better microhardness, but the microhardness of the spot weld zone gradually decreases as the welding speed increases, eventually falling below that of PDSP = 0.5 mm.

## 5. Discussion

The above conclusions were derived under the process parameters defined in this study. However, it should be noted that prior to this study, exploratory tests were conducted with a PDSP of 1.5 mm. The results indicated that while good-shaped weld zones could be obtained, the stirring sleeve at a point welding pressure of 7 KN did not adhere to the material surface, leading to some metal being extruded from the weld zone to form a protrusion, and significant bending deformation occurred around the weld zone, which is clearly an undesirable outcome. This also confirms that when PDSP is less than 1 mm in this study, it significantly increases the axial pressure and metal flowability in the weld zone, as the volume of metal that can be compressed is limited. Additionally, too large a PDSP causes the formation of too deep a depression on the surface of the weld zone, which conflicts with the technical characteristics of RFSSW and is also not very suitable for practical engineering applications.

Therefore, RFSSW with PDSP remains an effective means to improve the quality of the weld zone, as increasing the pressure in the weld zone can enhance the forging effect, which is beneficial for grain refinement and improving the density of the weld zone. Especially for materials prone to defects within the weld zone, such as dissimilar material welds where the mixing ability is limited, using a process form with PDSP may be a good solution. This method does not require any changes to the stirring tool; it only requires adjusting the relative position of the stirring pin and does not affect the actual welding time.

## Figures and Tables

**Table 1:** Chemical composition content of 6061-T6 aluminum alloy.

Each Element’s Composition (wt) %								
Si	Fe	Cu	Cr	Mg	Mn	Ti	Zn	Al
0.65	0.56	0.28	0.2	1.08	0.052	0.019	0.01	Others

**Table 2:** Mechanical properties and physical properties of 6061-T6 aluminum alloy.

Tensile Strength/MPa	Elongation/%	Hardness/HV_0.3_	Average Grains/μm
304	14	92	55

## Data Availability

The original contributions presented in this study are included in the article. Further inquiries can be directed to the corresponding author.
